# Alternative approaches to lymphoedema care in lymphatic filariasis

**DOI:** 10.1371/journal.pntd.0009293

**Published:** 2021-04-29

**Authors:** Suma Krishnasastry, Charles D. Mackenzie

**Affiliations:** 1 WHO Collaborating Centre for Lymphatic Filariasis Morbidity, Government TD Medical College, Alappuzha, Kerala, India; 2 Neglected Tropical Diseases Support Center, Task Force for Global Health, Atlanta, Georgia, United States of America; University of Zurich, SWITZERLAND

Caring for those clinically affected by lymphatic filariasis (LF) is increasingly being recognised as a central activity within the global effort to eliminate this debilitating infection. The World Health Organisation has specifically included this provision of care as an essential component of the global LF elimination program and as a requirement for achieving global success [1,2]. WHO recommends that a Minimum Package of Care (MPC) is made available to every LF patient with lymphoedema (LE) that includes the daily washing of the affected area, the use of topical creams for wounds and local infections, as well as the provision of therapeutic support for those suffering from the acute filarial attacks that commonly debilitate these patients [3]. The MPC, also known as the “essential care package,” is a relatively simple and field-applicable tool and is remarkably effective in rural and low-income communities [3,4]. Due to the relatively poor access to medical care in these areas, more sophisticated medical and complicated surgical procedures are often not available. Nevertheless, there have been efforts in different endemic locations around the world to include procedures additional to those recommended in the MPC for LE patients, such as vascular surgery and compression. In addition, in some countries, there are local customs such as puncturing the swollen limb with sharp instruments. It is important to ask whether or not all of these additional approaches are practical, are safe, and are appropriate means of improving the health and well-being of these patients.

Encouragingly, there are various ongoing efforts to improve the care available for LF-affected people, ranging from new LE assessment tools to newer topical and systemic treatments (Table 1). The usefulness of existing chemotherapeutic agents in stopping or reversing the development of lymphoedema is an important area for investigation. Doxycycline, which has been suggested to reduce lymphoedema [5], is undergoing testing in a 6-country multicentre trial for its effect on lymphoedema initiated by LF or by podoconiosis [6]. Likewise, the continuing use of the standard antifilarial agents used to break transmission of this infection has been seen to have a positive effect on LF-induced lymphoedema [7]. It has been generally thought that LF patients with lymphoedema no longer carry living filarial parasites, but this may not be entirely true as antifilarial agents do appear to a positive effect on patients’ lymphoedematous condition, at least in the early stages of treatment. The latter observations, taken together with the important Indian study of Shenoy and colleagues [8] that demonstrated that reversal of the pathology of lymph vessel dilatation occurs when the causative parasite is removed with the preventive chemotherapy drugs, support the notion that it is essential to ensure that no parasites remain in these patients. In recent years, interventions such as specialised massage, mental health, and rehabilitation have been considered as important additional components for LF patient care in some care programmes [9,10].

**Table 1:** Medical interventions additional to the WHO Minimal Care Package for filarial lymphoedema.

INTERVENTION	ISSUES	COMMENT
Anti-infection enhanced washing	Testing and availability	New anti-infective agents, often with healing promotion characteristics are available for testing
Antifilarial therapy with the MDA agents*	Not to be used during acute filarial attacks	May require modification of donated MDA drugs in post-MDA periods where there are still LE patients remaining
Antibiotic treatment	Availability	Doxycycline trials are under way [6]
Skin emollients	Local agents important—oils	Keeping natural skin moisture through simple oils, etc. is important in maintaining skin protection
Massage	Special methodology essential	Requires training, time consuming but north less useful when used
Wound care	Important	The availability of antibiotic creams etc. is an issue; new anti-infective and healing agents may help (e.g., HCLO**)
Improved assessment of dermal and tissue changes	The need to improve assessment of patients	It is important to investigate new approaches to assessment of the dermal tissue and vascular status (e.g., indurometry [20], thermal imaging [21], and digital volume assessment [22].
Reconstructive surgery	Not generally recommended	Field experience indicates a very poor outcome for these cases
Rehabilitation	An important component	It is important to consider not just the patient’s clinical condition but also their lives as a whole
Family care	Important and often ignored	An LE patient in family affects the family as a whole
Mental health	Important	An important recent addition to the understanding of LF patients and their care [9]

*MDA agents as used in the particular endemic area—ivermectin and albendazole in onchocerciasis-endemic regions; diethylcarbamazine and albendazole in other LF endemic areas (with the addition of ivermectin in certain areas).

**HCLO (hypochlorous acid has properties of microbial sterilisation and improving healing [20].

HCLO, hypochlorous acid; LE, lymphoedema; LF, lymphatic filariasis; MDA, mass drug administration.

Specific alternative interventions currently being used in some LF-endemic areas deserve special attention in this present discussion and, perhaps, specific concern. These include those that involve procedures that are invasive to these clinically affected, compromised limbs and tissues; a prime example here that of elective surgery to reduce limb bulk [11–13]. Various surgical methods are described in the literature, even from earlier days of LF patient care, for treating advanced stages of LE of the extremities using excisional procedures, and physiologic procedures such as node-venous shunt and lymphatico-venous anastomosis [14–16]. Vascularised lymph node transfer (VLNT) is thought to be promising technique used for the microsurgical treatment of extremity lymphedema in selected patients with moderate to advanced stage disease [17]. These surgically complicated approaches may indeed be useful in certain cases in advanced healthcare settings under experienced surgeons, but are arguably unsuitable for the large number of filariasis-induced cases that reside in medically underserved endemic areas.

Even though there is an immediate relief, surgical interventions in many LF-endemic areas in our experience almost always lead to worrisome outcomes, such as disfiguring healing and in some cases a need for amputation; the latter occurring often after long and distressing periods of poor response to the surgery. Two factors are likely to be contributing to these unsuccessful outcomes. Firstly, the fact that this surgery is being carried out on dermal tissues, supportive tissues, and vasculature that are essentially abnormal and compromised in terms of normal homeostatic mechanisms. Observations from the field suggest that there is a reduced ability of lymphoedematous tissues to heal in a regular manner; consequently, healing is poor with a predilection for the development of dermal keloids, multiple nodules, and uneven cicatrisation. Secondly, the compromised state of LE tissues underscores the need for heightened infection security in the surgical procedure and the follow-up period. These are issues that depend on a strong supportive health system which, unfortunately, is often lacking in many LF-endemic areas. This lack of needed medical support and adequate postsurgical care often leads to failed recovery from surgery and often to the limb amputation necessary to save these patients’ lives.

However, it should also be acknowledged that there are areas of the LF-endemic world where reconstructive surgeries on LE are still considered a very successful approach [11]; these reports for the most part involve LE patients in countries with relatively sophisticated medical systems and are not from rural areas of the endemic world where the long-term postoperative care for such patients is weak. In contrast to these reports of successful surgery, our own experience in India and Africa is that surgery of lymphoedematous limbs commonly does not lead to improvement in the lives of these patients.

In India, where there are 256 districts involved in the global program to eliminate LF, the basic essential care package does not include surgery for lymphoedema. However, many surgical centres in India do carry out reconstructive or debulking surgery for extensively lymphoedematous limbs due to LF. Although these patients do get relief and reduction in the oedema immediately after the surgery, problems commonly develop around 1 to 2 years after the surgery: The overall shape of the leg changes and skin develops abnormalities such as nodules and tightening and deformity of the skin (Fig 1). Nodules are a constant source for wounds or dermal erosions and are entry lesions for the secondary infections leading to recurrent acute systemic attacks. Thus, these patients’ quality of life turns from the welcome relief felt immediately after surgery to being even more miserable than before they had surgery—this often leads to the undesirable decision to request amputation of the affected limb. We have observed that the clinical follow-up for most LE surgical cases in India are on average only for the first year after surgery, and it is common that many of these unwanted clinical changes often develop beyond this time period.

**Fig 1:**
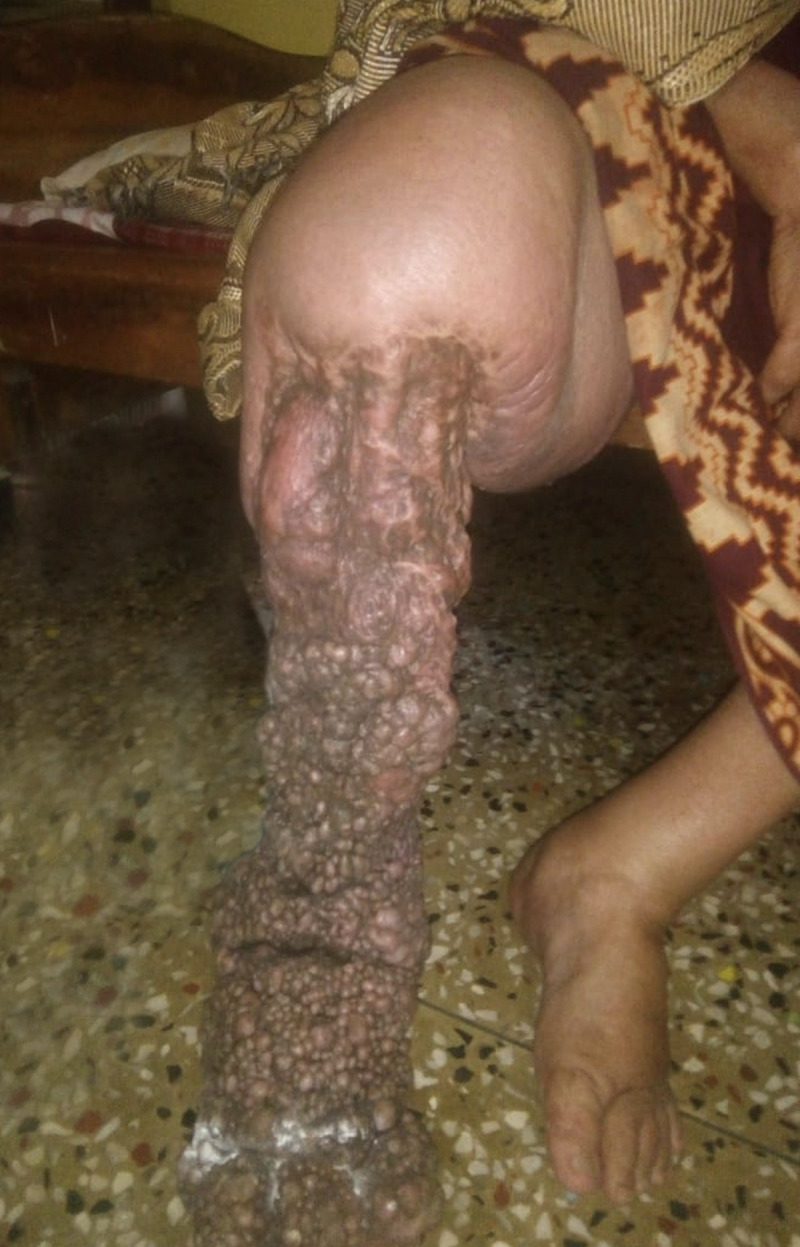
A 60-year-old female who underwent surgery to debulk the lymphoedematous tissue on her right leg 4 years previously; extensive postsurgery scarring, nodule formation, and dermal cicatrisation are evident.

In Tanzania, where in the past 20 years the national program has provided care for LF lymphoedematous patients, none of the 11 patients in the LF Programme’s original patient registry of over 1,000 LE cases who undertook LE reductive surgery had a satisfactory long-term outcome to their procedure; three required their affected limbs to be amputated. Consequently, the advice currently given to LF patients requesting surgery in Tanzania is to warn them against this procedure and that surgery should only be considered in rare and special clinical situations where it essential to saving their lives. In Tanzania, there is also a local “procedure” for treating lymphoedema that involves puncturing the swollen skin with a sharp instrument such as a nail or a knife; this most often results, not surprisingly, in a worsening of the condition, and commonly the development of keloiding scar lesions and tissue disfigurement. Happily, the rollout of the LF elimination program in Tanzania and the positive advocacy associated with the implementation of the MPC has greatly reduced this practice in the past ten years or so, and it is now rare to see patients that have suffered from this errant procedure.

It is important to emphasise here that the factors associated with failed LE surgical interventions described above do not apply to the essential surgical component of healthcare in bancroftian filariasis, namely hydrocoelectomy [18]. Surgical reduction of these hydrocœles, a very common healthcare activity of LF programs, is most often carried out on patients whose skin in the target surgical areas (the scrotum) is not commonly affected with lymphoedematous changes and thus will heal more regularly; adequate postoperative care nevertheless is still vitally important for surgical success. There are, however, cases where the skin of the scrotum is abnormal (elephantiasis of the scrotal dermal tissues, etc.), and the caution we urge above for surgery on LE limbs indeed also applies in these cases. Hydrocelectomy surgery nevertheless can be carried out when there is lymphoedema present in the scrotum; however, it is recommended to treat and reduce the lymphoedema first with standard procedures before carrying out the hydrocoele-reducing surgical procedure [18]. It should also be noted that plastic surgery on extensive disfigured male genital tissues has been carried out very successfully with filariasis patients, although these have been cases handled by expert surgeons in well-equipped medical settings [19].

Despite the advances in care for filariasis patients brought about by the instigation of the global filariasis elimination program [2], it is important that both new approaches to treatment and to the assessment of LE in filarial patients are developed. Recently, some newer techniques are being used to assess LE patients in filariasis-endemic areas taking the lessons from the procedures used in more advanced medical settings [20,21]: Infrared imaging, indurometry, and the detection of fluid content of the affected tissues are all now being implemented in early field studies, as is a more accurate means of measuring limb volume [22]. Likewise, new approaches to sterilising and physiologically supporting the affected dermal tissues with new approved sterilising agents [23] and other skin rehydration products are been instigated in endemic areas and are likely to improve even further the care provided to these once forgotten patients.

The experiences we describe here in this communication lead to our firm belief that invasive procedures in lymphoedema patients with their compromised tissues should be avoided wherever possible. Indeed, an essential component when caring for affected LE limbs and other affected areas is to attentively treat and heal all areas of damaged and abnormal skin (such as puncture wounds, ulcers, cracked heels, damaged interdigital skin and nail beds), and to move to restore the ability of the affected areas of skin to perform its normal protective function. In contrast to our negative opinion concerning surgery in any defined LE care package, we do however strongly support continuing the search for ways of improving care beyond the relatively simple components of the MPC. It should be emphasised that it is important not to give patients false hope of recovery, as undergoing LE surgery has done in many cases; these patients are in many cases already suffering from depression and self and external stigmatisation.

Fundamental to improving care for LF patients is the acquisition of a more detailed understanding of many different aspects of this complex condition and its medical management. This includes the following: the pathogenesis and progression of the condition, the optimal forms of physiotherapy and massage, the use of compression bandages, the mental health aspects of this condition, the importance of rehabilitation, as well as the effect an LE patient has on their families and caregivers. These are areas that if addressed through specific adequately controlled analysis will in all likelihood lead to improved care available for the more than 30 million LF lymphoedema patients that remain in the world today. It is vitally important that we continue as a healthcare community continue to seek for better ways of providing care for these patients. Finally, it is hoped that as the global LF elimination program reaches success in reducing transmission of this infection, there will be a marked reduction in new LE cases. With appropriate treatment being initiated earlier in the course of the developing condition, there will be less and less need for an LF lymphoedema patient to even consider major interventions such as surgery in the first place.

## Ethics statement

Written approval for the taking and use of the photograph in Fig 1 was obtained from the legal guardian.
